# Investigation of time–temperature dependency of heat capacity enhancement in molten salt nanofluids

**DOI:** 10.1039/d0ra03666h

**Published:** 2020-06-16

**Authors:** Syed Muhammad Mujtaba Rizvi, Baha El Far, Yousof Nayfeh, Donghyun Shin

**Affiliations:** School of Engineering & Technology, Central Michigan University Mount Pleasant MI 48859 USA shin1d@cmich.edu

## Abstract

In this study, the time–temperature dependency of heat capacity enhancement in molten salt nanofluids was studied experimentally. The result shows the heat capacity enhancement is directly related to the time-dependent synthesis process. Various samples of a binary salt mixture of Li_2_CO_3_–K_2_CO_3_ doped with 1% Al_2_O_3_ were prepared by heating and cooling at different rates (2, 4, 6, 8, and 10 °C min^−1^) along with the pure binary salt mixture. The samples were then tested for heat capacity using a differential scanning calorimeter. It was found that heat capacity enhancement in molten salt nanofluids depends on the heating and cooling rates during the synthesis. Recent studies have shown that the heat capacity enhancement observed could be due to the formation of dendritic structures. Transmission electron microscopy (TEM) and a pH variation method were employed to confirm the presence of dendritic nanostructures.

## Abstract 2

In this study, the time–temperature dependency of heat capacity enhancement in molten salt nanofluids was studied experimentally.

## Graphical abstract

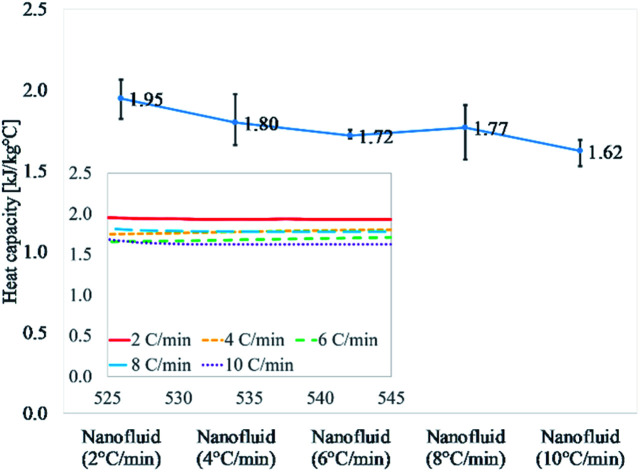

## Introduction

Concentrated solar power (CSP) is an emerging renewable energy production technology. Its working principle lies in the conversion of widely scattered solar energy into electrical energy using various thermodynamic cycles (*e.g.* Rankin, Sterling). Therefore, its efficacy and yield are in direct relation to the thermal storage and transport system incorporated within these plants. The materials and methods employed in these thermal systems have an enormous effect on the cost, net yield, management, and production of these plants. Conventionally, organic oils, stable up to 400 °C, are used in these plants for heat transfer.^4^ However, other attractive options such as eutectic molten salts are being effectively employed due to their high-temperature stability. A diligent study of thermo-physical properties and exploration of methods of advancement and elucidating the mechanism underlying heat capacity enhancements in molten salt nanofluids can greatly impact the functioning and performance of concentrated solar power.

Stable colloids of nanoparticles in a solution are defined as “nanofluids”. These are usually obtained by stabilizing very low mass or volume concentrations of nanoparticles in base fluids. Since their advent in the early 1990s,^5^ their thermodynamic properties, such as thermal conductivity has been studied extensively by researchers. These studies advocated large enhancement in thermal conductivities which were not in agreement with classical theories.^6–20^ However, researchers have come up with various theories in time to explain the anomalous behavior of these enhancements.^11–20^ At first, Brownian motion was deemed to be solely responsible for such behavior but did not get much attention.^11,12,16^ Similarly, the effect was tried to be explained using liquid layering around solid particles^13,14,17^ and thermal resistance between the interface theory was also established and tested experimentally and was found quite comprehensive. However, agglomeration of nanoparticles in conjecture with the effective medium theory is also reported in good relationship with practical results.^8,20^

Despite over numerous time and effort has been given to enhance and describe thermal conductivity in nanofluids, the studies on the specific heat of nanofluids were not given much attention as conventional nanofluids such as water-based, ethylene glycol-based nanofluids do not give good specific heat enhancement.^21–23^ It is often deemed that thermal conductivity is a single most important parameter to describe the efficiency of a dynamic operating system, but specific heat is more related to the system in not only its own quantity but also the sizes of associate thermal transport and storage systems. Early studies in nanofluids were mostly water-based metal or metal oxide mixture^20^ with a little exception of organic glycol-based nanofluids. However, it was Nelson *et al.*^24^ who firstly showed enhancement in specific heat when nanoparticles are doped in a mixture of two or more base fluids. Since then, several kinds of research have been reported for large enhancement in the specific heat of eutectic mixture-based nanofluids, mainly molten salt mixtures including binary/ternary nitrate salt mixtures, binary/ternary carbonate salt mixtures, and chloride salt mixtures.^1,3,25–41^

Although enormous experimentation was carried out to show enhancement in the specific heat of molten salt nanofluids, the mechanism behind the enhancement is yet to be fully understood. Several attempts have been made to explain the enhancement in specific heat. They include (1) higher specific heat capacity of nanoparticles, (2) solid–fluid interaction energy, (3) “layering” of liquid molecules at the surface to form a semi-solid layer.^26^ However, the low concentration of nanoparticles makes it hard to believe any of these mechanisms dominates the observed thermal storage phenomena. Among other recently reported mechanisms, it was understood by recent studies that the observed enhancement in specific heat in the literature might result from dendritic nanostructure formed in the salts,^1,3,28,40^ which could be induced by electrostatic interactions between salts and nanoparticles.^1^ Later on, it was found that these nanostructures can be formed by dendritic formation in nature by crystallized salt grown on the surface of the dispersed nanoparticles.^1,3,40,42–45^

The discussion becomes further inconclusive with the addition of the fact that a huge discrepancy of the results was observed for the same molten salt nanofluid system.^46^ However, most of these researches were carried out with the same sample preparation (Shin and Banerjee method^26^) which rules out that this variation is coming from sample preparation alone and we must look for other possibilities to elucidate the phenomena causing this. A detailed literature review shows that there is a quite large variation in their measurement methods. Some used standard DSC (ASTM E1269) method, which typically uses a very fast ramping rate of 10 °C min^−1^ to 20 °C min^−1^ to scale the heat flow signal to a sample in the DSC.^25–28^ On the other hand, later researches used a much slow ramping rate of 2 °C min^−1^ or 3 °C min^−1^ with advanced modulation technique in MDSC.^3,41^

This advocates a strong need to reiterate this variation in an organized manner and observe the variation trend which could be studied considering existing variation models. One of the potential candidates behind the mechanism could be the thermophoresis effect. The thermophoresis effect is, widely known phenomena in material science and mechanical engineering, which describes the movement of different particles in Brownian motion in correlation with thermal gradient.^47^ The discussion of the thermophoresis effect in the nanofluid area is not new. It has been studied by various researchers to explain enhanced thermal conductivity and other phenomena.^47–50^ Sheikholeslami *et al.*^49^ used the thermophoresis effect to study the movement of Al_2_O_3_ nanoparticle in water. The thermophoresis effect could be very crucial in explaining the movement of different types of ions in a molten salt nanofluid system where salt molecules can crystalize around nanoparticles. This may cause localize change in the composition of salt causing hypo or hyper eutectic compounds to form which can survive past the melting point of the eutectic mixture as observed in the reported experimental studies.^1,3,40,41^

Further, as we are dealing with the crystallization of solids. It is important to look at the nucleation rate and grain growth of these structures too. Thermophoresis may describe the dynamics of nanostructure formation but to understand its kinetics it is important to discuss the role of nucleation and grain growth about it as well. Nucleation is the formation of solid nuclei in the liquid pool through which a solid could grow into a grain size.^51^ Our case is strictly a case of heterogeneous nucleation, in which a solid begins to grow on foreign particles.^52^ It is widely observed phenomena in literature as well that heterogeneous nucleation is observed. When foreign particles are present within a eutectic colloidal system.^52–55^ As these nucleation and grain growth are affected by temperature gradients and time provided,^53,54^ we must discuss nucleation and grain growth of nanostructure in correlation with thermal gradient and time provided for growth. To prove this hypothesis, we employed multiple thermal cycling protocols to test different heating conditions during the synthesis of molten salt nanofluids. In this way, it is hypothesized that nucleation and growth of dendritic nanostructure can be different and the resultant heat capacity enhancement can be changed. Also, we performed Tiznobaik's technique^40^ to verify the effect of the structure on the resultant heat capacity enhancement, as an indirect experimental tool to observe the change of the dendritic structure formation because it takes place at extremely high operation temperatures (over 500 °C) and moisture-free environment. A transmission electronmicroscopy was also employed to characterize the salt dendritic nanostructures.

## Method

### Sample preparation

Salt preparation technique as described by the literature were used.^40^ Li_2_CO_3_ & K_2_CO_3_ were procured by Acros Organics with 99.99% purity. Al_2_O_3_ nanoparticles were procured by Meliorum Tech Inc. (Rochester, NY). The particle size distribution analysis has been performed by a photon correlation spectroscopy (PCS; Beckman Coulter N4 Plus) and it was shown in the result that the mean size is 10.8 nanometers with a standard deviation of 4.1 nanometers (Table 1).

**Table 1:** Particle size distribution analysis by photon correlation spectroscopy (Beckman Coulter N4 PLUS Particle Size Analyzer)

Angle	Sampling time	Counts	Baseline error	Intensity	Mean	STDEV
90°	2.5 μs	1.5	0.18%	6.648 × 10^5^	10.8 nm	4.1 nm

First of all, a pure binary eutectic mixture of Li_2_CO_3_ and K_2_CO_3_ was prepared by mixing them in the mole ratio of 62 : 38, respectively. A total of 198.00 mg containing 92.11 mg of Li_2_CO_3_ and 105.88 mg of K_2_CO_3_ were completely dried on a hot plate for hours and precisely measured in a microbalance (SECURA225D, Sartorius) and mixed in 25 ml vial in solid-state. 20 ml of water was then added to it. The mixture was then mixed for 2 hours using BRANSON 5200 sonicator. The mixture was then heated at 200 °C for 20 minutes to remove moisture and then at 300 °C for 200 minutes for removing any bonded water molecules in a 10 cm Petri dish. The dried sample was then further mixed manually using a spatula to ensure homogeneous mixture. Nine different samples of mass between 9–10 mg were sealed in hermetic aluminum pan. The samples were then heated and cooled (thermally cycles) between 350 °C and 550 °C. Temperature range is provided to heat and cool salt across its melting point (490 °C (ref. 25)) at 2, 6, and 10 °C min^−1^ using differential scanning calorimeter (TA instrument, DSC 25). Three samples at each cycling rate. Similarly, a binary carbonate mixture with the same ratio was preheated on a hot plate and measured using the same physical balance. 2 mg of Al_2_O_3_ was preheated on a hot plate and then added to the salt. Salt and nanoparticles were poured in a 25 ml vial in solid-state. 20 ml of water was then poured into it and sonicated for 2 hours using BRANSON 5200. This sample was also dried at first at 200 °C for 20 min for removal of water and then at 300 °C for 2 hours. 15 samples were sealed in a hermetic aluminum pan with a mass between 9–10 mg. Samples were then thermally cycled to at different heating rates at 2, 4, 6, 8, and 10 °C min^−1^. Three samples with each cycling rate in the same range as of pure sample in the DSC mentioned above. Another batch of salt was prepared in the same ratio as above, 2 mg of Al_2_O_3_ nanoparticles and 0.02% NaOH were doped and the mixtures went through the same procedure of wet mixing, ultrasonication, and drying. These three samples were thermally cycled at 2 °C min^−1^ (which later confirmed to show the highest heat capacity enhancement) *via* the same DSC. This sample was made to verify the formation of the dendritic nanostructure, we followed the same verification experiment reported in the literature.^40^ All these 27 samples were prepared and tested for heat capacity measurement in a cleanroom environment to ensure no contamination from airborne particles.

### Heat capacity measurement

The heat capacity of each sample was characterized by a differential scanning calorimeter (DSC 25, TA Instruments) using the standard heat capacity measurement protocol (ASTM E-1269 method). Constant environment was made to be provided by controlling humidity below 20% within the workplace, and maintaining the mass of each sample in the range of ∼9–10 mg to minimize the potential effect on the heat flow inside DSC cell. Tested masses of all samples were within a range of 1 mg from each other. All the testing procedure took place in a cleanroom environment to ensure no airborne particle contamination in the sample preparation. Heat capacity was measured between 460–550 °C at 20 °C min^−1^ rate.

### Melting point

Melting point assessment was also performed for all samples prepared by heating/cooling (thermally cycling) them at temperature rates. The melting point of the pure eutectic mixture at three different rates of *i.e.* 2, 6, and 10 °C min^−1^ and nanofluids at five different rates *i.e.* 2, 4, 6, 8, and 10 °C min^−1^ was measured. Melting point of a sample containing 1% Al_2_O_3_ and 0.02% NaOH thermally cycled at 2 °C min^−1^ was also measured. Onset temperature was measured using well-defined techniques in literature.^56,57^ Between peak and onset temperatures, onset temperature was chosen to determine the melting temperature because peak temperature can be affected by different heating rates.^56^ The onset melting temperature, *T*_onset_, is defined as the point of intersection of the extrapolated base line of the DSC curve and the tangent of the principal side of the melting peak. The equations of both lines were calculated by linear extrapolation and the point of intersection (*i.e. T*_onset_) was found accordingly.

### Material characterization

Material characterization was performed using transmission electron microscopy (Hitachi HT 7700). Samples from pure Li_2_CO_3_–K_2_CO_3_ and its nanofluids thermally cycled at 2 °C min^−1^ and 10 °C min^−1^ were dissolved in 200 proof ethyl alcohol for dilution while minimizing exposure to moisture. The samples were then poured onto to carbon-coated copper grid and the grids were then imaged using Hitachi HT 7700 Transmission electron microscopy. 100 kV voltage was applied between cathode and anode to avoid transmission of excessive energy and possible ionization of samples. The beam height and alignment were adjusted at 50 000× and all imaging was carried out below this magnification. This was done to avoid any diffraction resulting in shadow formation of particles that may look like nanostructure. 300 nm objective aperture was used to avoid stigmation. All images were taken in the high-resolution mode for better sizing and observation of structures.

## Results & discussion

### Heat capacity measurement

Table 2 discusses heat capacity results of pure eutectic mixture. It comprises of heat capacity results of 9 samples thermally cycled at different heating rates. Three samples cycled at 2 °C min^−1^, three at 6 °C min^−1^, and three at 10 °C min^−1^ repeated over three times. The average results for 2, 6, and 10 °C min^−1^ are 1.57 kJ kg^−1^ °C^−1^, 1.58 kJ kg^−1^ °C^−1^ and 1.59 kJ kg^−1^ °C^−1^ respectively. All these results are in close agreement with the literature value of 1.60 kJ kg^−1^ °C^−1^.^25^ These results show two important matters. There is no significant heat capacity change for heating/cooling (thermal cycle) used. The error from the measurements was not significant. The random error was calculated by *t*_0.95,*k*_ × *S*_*x̄*_/*x̄*, where *t*_0.95,*k*_ is student *t*-value for a confidence probability of 0.95 and degrees of freedom *k* = *n* − 1, where *n* is number of measurement. *S*_*x̄*_ is variance and *x̄* is average value. The random uncertainty of all 27 measurements is only 0.67%.

**Table 2:** Heat capacity of pure binary carbonates prepared over rates (2, 6, and 10 °C min^−1^), measured by a differential scanning calorimeter (DSC 25, TA Instruments) at 540 °C. The unit is kJ kg^−1^ °C^−1^

Sample #	Test #	2 °C min^−1^	6 °C min^−1^	10 °C min^−1^
1	1	1.53	1.63	1.57
	2	1.53	1.63	1.57
	3	1.53	1.59	1.57
2	1	1.59	1.57	1.62
	2	1.58	1.57	1.60
	3	1.58	1.56	1.61
3	1	1.6	1.55	1.60
	2	1.6	1.56	1.60
	3	1.59	1.56	1.57
Average		1.57	1.58	1.59
Standard deviation		0.03	0.03	0.02

**Fig. 1:**
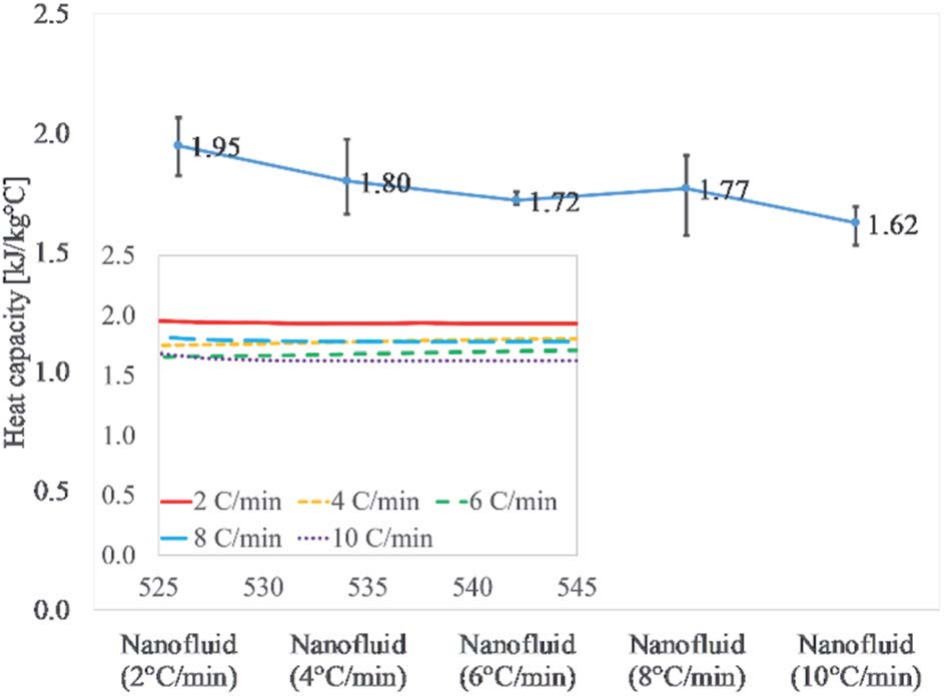
Heat capacity of samples prepared at different heating rates (2, 4, 6, 8, and 10 °C min^−1^). It shows a declining trend with increasing the ramping rate at 540 °C.

Fig. 1 and Table 3 discuss the results of nanofluids thermally cycled at different rates. This comprises of heat capacity results of 15 different samples repeated three different cycles. The obtained average value for 2 °C min^−1^ is 1.95 kJ kg^−1^ °C^−1^ which is the highest of all results. As the heating/cooling (thermal cycle) rate increases the value of heat capacity begins to drop. The heat capacity values for the next three heating rates, 4, 6, and 8 °C min^−1^ are 1.80, 1.72, and 1.77 kJ kg^−1^ °C^−1^, respectively. The heat capacity value for the highest heating rate of 10 °C min^−1^ is the lowest at 1.62 kJ kg^−1^ °C^−1^. This describes a declining trend in heat capacity enhancement of molten salt nanofluid with an increasing thermal cycling rate during the syntheses. The random error using a confidence probability of 0.95 is 5% or less for all the five different heating rates of 2 to 10 °C min^−1^.

**Table 3:** Heat capacity of nanofluid samples prepared at different heating rates (2, 4, 6, 8, and 10 °C min^−1^), measured by a differential scanning calorimeter (DSC 25, TA Instruments) at 540 °C. The unit is kJ kg^−1^ °C^−1^

Sample #	Test #	2 °C min^−1^	4 °C min^−1^	6 °C min^−1^	8 °C min^−1^	10 °C min^−1^
1	1	2.04	1.77	1.72	1.72	1.69
	2	1.99	1.74	1.72	1.67	1.69
	3	1.89	1.66	1.71	1.57	1.53
2	1	1.96	1.97	1.70	1.91	1.64
	2	2.06	1.94	1.71	1.91	1.63
	3	1.99	1.90	1.71	1.89	1.58
3	1	1.82	1.78	1.76	1.78	1.61
	2	1.91	1.72	1.70	1.75	1.62
	3	1.87	1.69	1.71	1.71	1.61
Average		1.95	1.80	1.72	1.77	1.62
Standard deviation		0.08	0.11	0.02	0.12	0.05

**Fig. 2:**
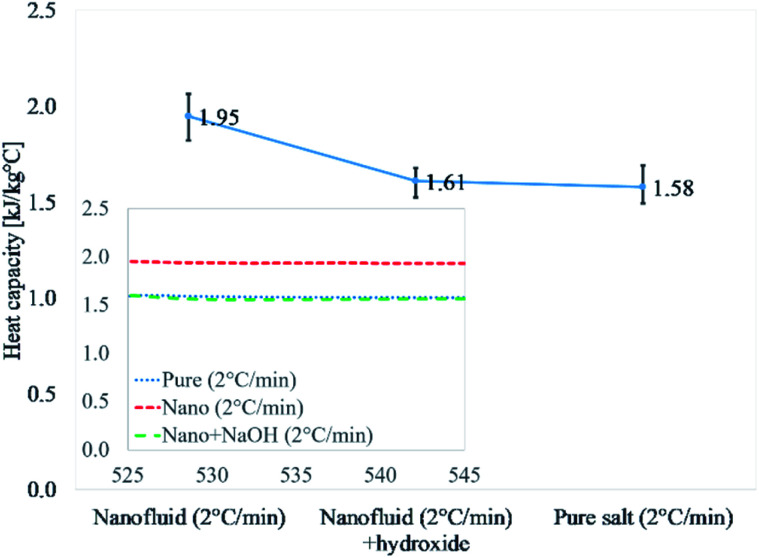
Experimental verification of dendritic structure formation in the same procedure in the literature.^40^ Values reported are at 540 °C.

Fig. 2 and Table 4 discuss the results of Li_2_CO_3_–K_2_CO_3_, pure eutectic mixture doped with 1% alumina Li_2_CO_3_–K_2_CO_3_–Al_2_O_3,_ and doped eutectic mixture with 0.02% NaOH. Li_2_CO_3_–K_2_CO_3_–Al_2_O_3_–NaOH was thermally cycled at 2 °C min^−1^ as the highest heat capacity enhancement is reported for 2 °C min^−1^ (Table 3). The results for nanofluid were 1.95 kJ kg^−1^ °C^−1^, whereas, those nanofluids doped with NaOH (0.02%) showed 1.61 kJ kg^−1^ °C^−1^. It means the addition of NaOH was able to disrupt the formation of salt dendritic nanostructures as reported in the literature.^40^ The degradation of the heat capacity for the sample containing NaOH is an indirect verification that salt dendritic nanostructure failed to form. The presence of salt dendritic nanostructures was confirmed by a transmission electron micrograph later.

**Table 4:** Heat capacity of pure Li_2_CO_3_–K_2_CO_3_, nanofluid (Li_2_CO_3_–K_2_CO_3_–Al_2_O_3_ (1 wt%)), and the nanofluid doped with hydroxide (Li_2_CO_3_–K_2_CO_3_–Al_2_O_3_ nanoparticles (1 wt%)–NaOH (0.02 wt%)). The result agreed well with the literature that the heat capacity enhancement diminished with the addition of NaOH,^40^ measured at 540 °C. The unit is kJ kg^−1^ °C^−1^ and all the samples were synthesized at 2 °C min^−1^

Sample #	Test #	Pure	Nanofluid	Nanofluid + hydroxide
1	1	1.49	2.04	1.59
	2	1.56	1.99	1.58
	3	1.56	1.89	1.68
2	1	1.53	1.96	1.64
	2	1.52	2.06	1.66
	3	1.55	1.99	1.68
3	1	1.67	1.82	1.55
	2	1.63	1.97	1.55
	3	1.69	1.87	1.52
Average		1.58	1.95	1.61
Standard deviation		0.02	0.03	0.02

Table 5 shows the onset temperatures for pure eutectic, nanofluid, and nanofluid containing 0.02% NaOH synthesized at different heating/cooling (thermal cycling) rates. Onset temperature for all systems was in strong agreement with the literature value of 490 °C.^58,59^ The maximum deviation from the reported value does not exceed more than 2.5 °C on either side. The standard deviation for pure samples at different rates is only 0.38 °C, and 1.77 °C for nanofluids. The low values of standard deviation show there no significant variation between salts prepared at different cycling rates. All onset temperatures recorded in this study also lies in the same range with small variations. This shows that neither the addition of constituents nor preparation method (heating and cooling at different rates before testing) significantly impacts the salt composition. Moreover, some recent studies^60,61^ have shown that dehydrating salt in different molten salt nanofluid preparation can result in a change of salt composition that may cause heat capacity variation. The result confirms that no significant change in salt composition occurred during the syntheses.

**Table 5:** Melting onset temperatures of pure eutectic mixture, eutectic mixture doped with 1% Al_2_O_3_ and doped eutectic mixture with 0.02% NOH prepared at different heating rates

Preparation rate	Pure salt	Nanofluid	Nanofluid + 0.02% NaOH
2 °C min^−1^	488.81 °C	488.01 °C	488.60 °C
4 °C min^−1^	—	489.30 °C	—
6 °C min^−1^	489.02 °C	492.66 °C	—
8 °C min^−1^	—	490.36 °C	—
10 °C min^−1^	488.28 °C	491.11 °C	—
Standard deviation	0.38 °C	1.77 °C	—

**Fig. 3:**
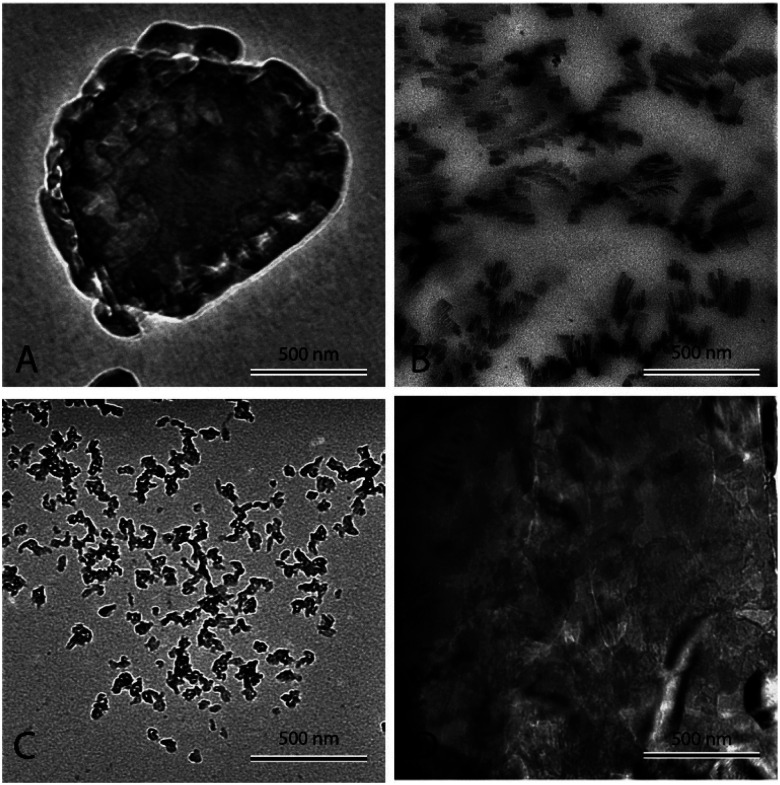
TEM micrographs of different salt systems. (A) Pure binary carbonate eutectic mixture. (B) Eutectic mixture doped with 1% Al_2_O_3_ thermally cycled at 2 °C min^−1^ before testing. (C) Eutectic mixture doped with 1% Al_2_O_3_ thermally cycled at 10 °C min^−1^ before testing. (D) Eutectic mixture doped with 1% Al_2_O_3_ and 0.02% NaOH thermally cycled at 2 °C min^−1^ before testing.

Fig. 3(A) shows pure eutectic binary carbonate prepared a 2 °C min^−1^. The micrograph shows bulk salt in the figure. It can be seen that no structure or any sort of assimilation can be seen in the figure. The salt looks plain and the heat capacity measurement (Table 2) does not show any heat capacity enhancement. Fig. 3(B) shows eutectic binary carbonate doped with 1% Al_2_O_3_ prepared by thermally cycling at 2 °C min^−1^. The figure shows a very well developed dendritic structure. It can be seen that the structure is elongated and very fine. Also, it springs in to further secondary and tertiary dendrites. This sample shows very high heat capacity enhancement (Table 3). The average value obtained for it is 1.95 kJ kg^−1^ °C^−1^. Fig. 3(C) is the same mixture as of Fig. 3(B) but this sample is prepared by thermal cycling at the fastest heating rate of 10 °C min^−1^. No proper or organized structure formation can be seen in the micrograph. However, it seems that structure tried to form but failed. Therefore, this sample does not show significant heat capacity enhancement (Table 3). The average value obtained for this system is 1.62 kJ kg^−1^ °C^−1^. Fig. 3(D) shows a TEM micrograph of eutectic carbonate doped with 1% Al_2_O_3_ and 0.02% NaOH. This sample also does not show any structure formation and agree well with the literature.^40^ This sample also failed to show any heat capacity enhancement (Table 4).

**Fig. 4:**
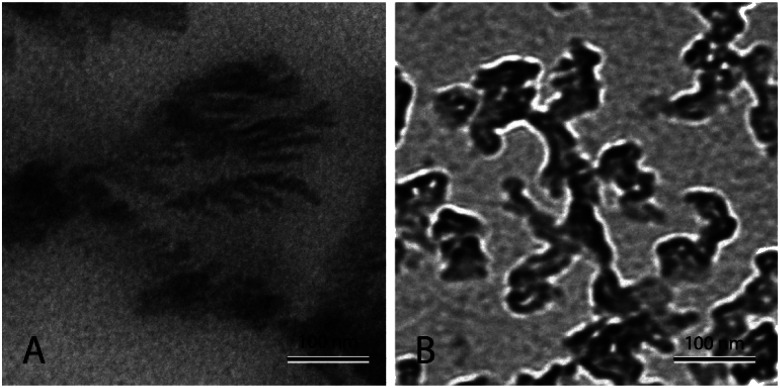
TEM micrograph showing molten salt nanofluids. (A) Nanofluid prepared by thermally cycle at 2 °C min^−1^, (B) nanofluid prepared by thermally cycle at 10 °C min^−1^.

Fig. 4(A) shows nanofluid heated and cooled at 2 °C min^−1^ between 460 °C and 550 °C during the synthesis. The figure shows that there exists a well-developed nanostructure. It shows formed dendrite in the center, which further elongates into secondary and even ternary dendrites. These structures are of the order of 100 nm or less. This sample showed a very high enhancement. On the other hand, Fig. 4(B) shows the nanofluid thermally cycled at 10 °C min^−1^ during the synthesis. The TEM micrograph shows that much thicker dendritic structure were formed. In comparison to Fig. 4(A), it can be seen that structure is not as well developed, which supports the fine salt dendritic nanostructure could be primarily responsible for the observed heat capacity enhancement. It elucidates the difference between structure formation when molten salt nanofluids are thermally cycled at different heating rates. They give us a hint that there exists time–temperature dependency in the heat capacity of these fluids. The amount of time and temperature provided to these salt plays a role in determining the heat capacity of enhancement. With the help of these images, it can be linked that time and temperature provided may determine the formation and abundance of these structures, which may result in the heat capacity enhancement.

### Presence of dendritic nanostructure and its effect on heat capacity

Dendritic structures, as the name suggests are very fine elongated developed solid structures. Several studies have explained in detail how temperature varies between the nanoparticles and the surrounding media, developing a temperature gradient between the two.^11,14,62^ However, in this case, the surrounding media is water, which has a very low ability to crystallize or order itself, only a mono-atomic liquid layer around the particle in the liquid phase is possible and not beyond. However, he left a blank for the possibility that other materials may behave differently around the nanoparticles.^14^ Later, Shin *et al.*^1^ proposed that the dendritic structure is formed by crystallized salt molecules near nanoparticles and may enhance the effective heat capacity (Fig. 3 (ref. 63)). Tiznobaik and Shin^40^ verified the effect of such nanostructures on the effective heat capacity of molten salt-based nanofluids experimentally. However, the growth mechanism was undiscovered.

Tiznobaik^40^ has already explained how heat capacity increases with the formation of a dendritic structure. According to the effective heat capacity of mixtures, the heat capacity is given by:1
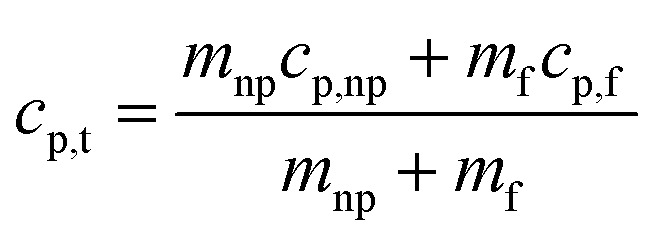
where *c*_p_ is specific heat and *m* is mass. Subscripts t, np, and f denote nanofluid, nanoparticle, and base fluid, respectively. However, in case of the presence of dendritic structure, the system is no longer binary and another effective heat capacity measure *i.e.* nanostructure must be added to the equation to explain the heat capacity enhancement. Therefore, eqn (1) must be re-written as:^1^2
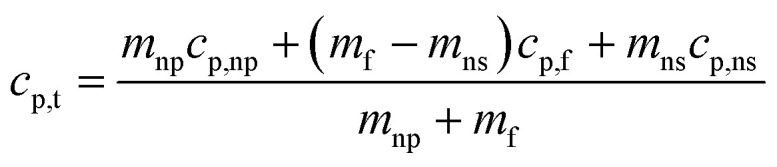
where subscript ns denotes salt dendritic nanostructures.

**Fig. 5:**
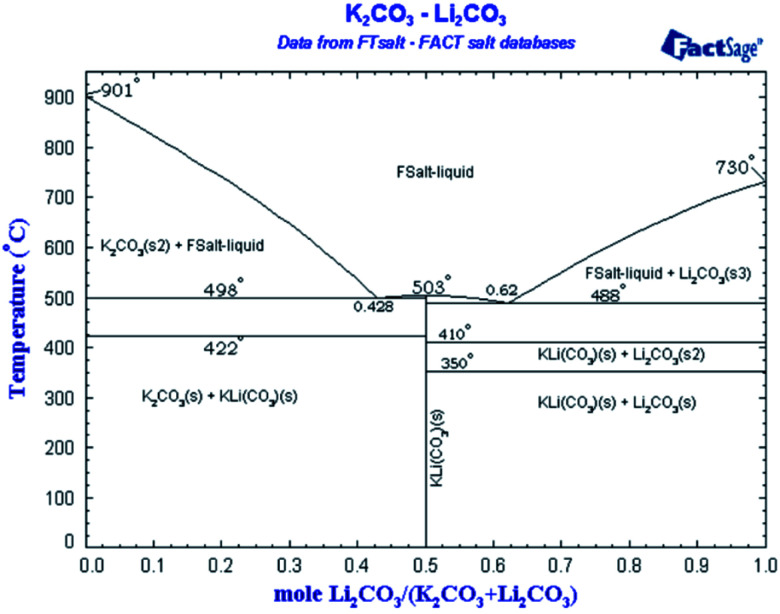
Binary phase diagram of Li_2_CO_3_–K_2_CO_3_.^64^

Regarding the presence of nanostructure in a molten state, so far there is no direct method to confirm the presence of these structures in the molten state due to very high temperatures. Therefore, we used a reported technique (NaOH test^2^) as an in-direct method to confirm the presence of dendritic structures in a molten state as shown in Fig. 2 and Table 4. Moreover, how such dendrites remain upon salt melting is already explained in the literature.^1^ Fig. 2b in ref. 10 is a backscattered electron image, which distinguishes different material or composition by contrast. The image shows the composition of salt dendrite has been completely changed from the eutectic point. It means the salt dendrite exists as either hypo-eutectic or hyper-eutectic. According to the phase diagram of Li_2_CO_3_–K_2_CO_3_ in Fig. 5,^64^ either hypo-eutectic or hyper-eutectic exists as a mixture of solid salt and liquid salt above the melting point (*i.e.*, 500 °C), where the solid salt results in the salt dendrites nucleated on the surface of nanoparticles. However, it should be maintained that this shifting from the eutectic ratio is out of microsegregation and does not affect the overall chemistry of the salt. The change is very localized and does not affect the melting temperature or behavior of overall salt. The phenomena are widely discussed in material science has been explained in detail how solid and liquid can co-exist without evident change to equilibrium point by Avner,^65^ Callister,^51^ and Fleming.^66^

### Possible cause of dendritic structure → micro-segregation

A mixture consists of more than one type of material. A homogeneous mixture is understood as a mixture in which phases cannot be distinguished separately. However, even a homogeneous mixture is not always homogenized and there can occur some variation of concentration at both micro- and macro-level. When a mixture shows segregation at atomic- to micro-level, it is called micro-segregation.^67^ This segregation may play a very critical role in defining localized chemistry, crystallography, grain orientation and grain size of nucleating constituent. This micro-segregation can increase or decrease the concentration of a constituent of the mixture and move its ratio from eutectic compound to hypo- or hyper-eutectic compounds.

### Possible causes of micro-segregation → electrostatic interaction & thermophoresis

The thermophoresis effect describes the motion of ions, particles or molecules present under a thermal gradient.^68^ If a particle is present in a close proximately to a surface that is hotter or colder than it, it will experience a force called “thermophoresis” force. This force may pull or push the particle towards it. Thermophoresis is defined as positive when a particle moves from the cold region to the hot region and negative when it moves from hot region to cold region.^68,69^ This movement depends on the size and charge of moving material and the steepness of gradient established.^70^ The effect already has an established position in this research area as well.^47–50^ Particularly, Sheikholeslami *et al.*^49^ study on alumina is important as we have the same nanoparticles. Shin *et al.*^1^ explained that nanostructures in a molten salt-based nanofluid could be a resultant of electrostatic attraction between nanoparticles and salt constituent. In a case of a binary molten salt-based nanofluid, one salt ion can be attracted more than the other salt ion to a nanoparticle causing localized changes in chemistry (*i.e.* micro-segregation^67^). However, the observed nanostructures in the reported experimental studies^1,3,40,41^ are quite significant just to assume the electrostatic interaction is a sole mechanism behind the formation of the dendritic nanostructure. It is possible that the micro-segregation does not occur by a sole cause but could be due to a combination of the thermophoresis and the electrostatic interactions. Unfortunately, distinguishing the effects of these two causes is not practically achievable in current available technologies due to extreme conditions of molten salt environments.

### Micro-segregation due to thermophoresis effect

As already explained by several researchers, there exists a thermal gradient between nanoparticles and media surrounding it.^11,14,62^ When heating Li_2_CO_3_–K_2_CO_3_-based Al_2_O_3_ nanofluids, different types of ion (K^+^, Li^+^, CO_3_^2−^) would move toward nanoparticles with different rate due to thermophoresis effect and it is possible that one salt type reaches nanoparticles more than the other salts, it is also possible some particles remain stagnant around nanoparticles and other migrate further away from them. All in all, there would be a very localized change in the composition of salt at nanoscale, resulting in the salt formation of a hyper or hypo eutectic compound which would be essentially solid according to salt phase diagram. However, it is important to focus that this segregation is strictly micro and is not observed on the macro level. Nanoparticles are not altering the whole chemical nature of the systems as observed in another shifting of the eutectic point overall. Macro-segregation would have caused a higher deviation of the eutectic point. This is mainly because the concentration of nanoparticles is too small (1% by weight in the present study) to produce any macroscopic change. The change is only localized and does not affect the overall system as the melting point is not shifted from the present study as well as previous research.^1,3,40,41^

### Dendritic structure formation: nucleation & growth

Any solid-state structure comes into formation with a combination of nucleation and then grain growth.^71^ These nucleation and grain growth phenomena define the shape and orientation of the solid material that came into existence.^68^ The processes are relatively simple for pure material for instance. Water. Upon cooling from the liquid state, the water molecules nucleate and grow into cubic crystals of ice.^71^ The phenomena start to get complicated for mixtures. Even for eutectic mixtures which behave as pure materials upon cooling melting. This can be viewed in the example of a eutectic compound of iron and carbon. Where the same eutectic composition of the mixture results in the fine laminar structure of perlite upon slow cooling,^69,70^ bainite upon moderate cooling^72^ and super fine-grained martensite upon very rapid cooling.^70^ Of course, all these materials have very peculiar physical and thermal properties despite the same chemical composition as properties depend on the molecular arrangement as well.^73–76^ Now let us see the dynamics of nucleation and grain growth in correlation with our study.

Nucleation processes in which solid material begins to crystalize in the liquid pool.^77^ This may occur homogeneously or heterogeneously^77,78^ in homogeneous nucleation, the material begins to crystalize by combing together and forming a chunk of solid molecules as we would see in water.^79^ Whereas, in heterogeneous nucleation, they begin to solidify on the surface of foreign present particles within the liquid pool. Our case is strictly heterogeneous nucleation as it is established by every previous research that these structures grow only on the surface of particles inducted.^1,3,40,41^ Let us see on what factors does this nucleation depends. For every nucleus which is coming into existence must reach a critical radius so it could be regarded as stable nuclei and growth only begin after it has reached critical nuclei.^80^ This critical nucleus is defined mathematically as:^81,82^3
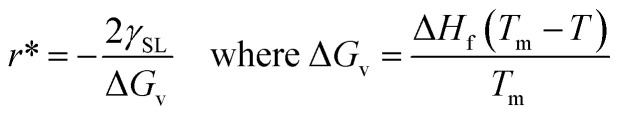
where r* is the critical radius, *γ*_SL_ is surface energy between solid and liquid, Δ*G*_v_ is the difference between free energy, Δ*H*_f_ is the heat of fusion, *T*_m_ is melting temperature. Since *γ*_SL_, Δ*H*_f_, and *T*_m_ can be considered as constants, we can rewrite r* in terms of *T* as follows:4
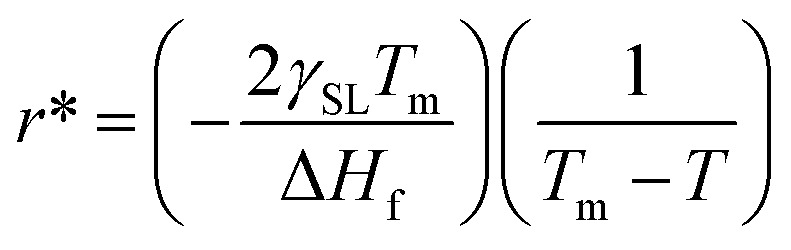


Eqn (4) shows that the radius obtained is inversely proportional to the difference in temperature from the melting point. This simply means that when the difference is very large the obtained radius is very low and most the nuclei would not reach the critical radius and will eventually dissolve back into the liquid pool. This is known as recoalescence.^79,83^ In other words, at the near melting temperature, the ease of reaching the critical radius is far easy for forming nuclei than for larger differences.

In the same manner, the kinetics of such a system can also be explained. For a sample length of time, a system moving at 2 °C min^−1^ (which showed the highest heat capacity enhancement) will go less far from melting temperature than 10 °C min^−1^ (which showed the least heat capacity enhancement). For example. After 10 min from letting temperature, the system would stand on *T*_m_ + 20 °C whereas, for 10 °C min^−1^ the system would stand at *T*_m_ + 100 °C. so a system ramping with 2 °C min^−1^ has a better chance for nucleation than at higher rates, giving rise to the abundant amount of nuclei and solid dendritic structure than as compared to higher rates. Nanodentrites form right after nucleation and grow very fast up to nano-metric lengths until after a very short distance they discontinue to grow any further.^84^

### Role of nucleation and grain growth to a salt dendritic nanostructure in our experimentation

The above discussion is the extensive elaboration of the time–temperature dependence of dendritic structure formation. However, it is quite evident from the above discussion that at 2 °C min^−1^ the system was given a reasonable enough time to nucleate itself during the synthesis and therefore could show greater effect on the heat capacity. On the other hand, at higher ramping rates during the synthesis, the system rapidly moves away from the melting temperature, giving it very less time to nucleate and grow and as a result, its effect on the heat capacity is limited. Going back to what has been explained to us by Tiznobaik,^40^ the contribution of salt dendritic nanostructure can be accounted for only if we use its heat capacity value and mole fraction while discussing the heat capacity of the overall system (eqn (2)). Unfortunately, how to measure the heat capacity and the concentration of the salt dendritic nanostructure is still questionable. However, our studies demonstrate that different heat and cooling (thermal cycling) rates during the nanofluid synthesis could affect the formation of the nanostructure and possibly be linked to heat capacity enhancement *via* this equation. When the ramping rate is low *i.e.* 2 °C min^−1^ the formation of structure is more is mole fraction and mass increases in the system, simultaneously decreasing the effect of base salt. This will result in a higher heat capacity. Whereas, at higher heating rates formation is less so is the effect on the heat capacity.

### Other possible mechanisms

In the given study, time–temperature dependence of heat capacity enhancement is studied, and it is found that using different heating rates give different heat capacity values. Here, we claim this enhancement could be linked to the formation of dendritic structures. The hypothesis is supported by the pH change validation study and the imaging by transmission electron microscopy. TEM images hint us nanostructure is well developed when samples are cycled at lower ramping rates. However, they can still be linked to other heat capacity mechanisms described in literature such as particle aggregation and liquid layering.^46,85–89^ For example, Mondragón *et al.*^90^ have proposed a very novel mechanism for heat capacity enhancement of molten salt nanofluids in terms of ionic exchange between nitrate ions of salts and silica nanoparticles. The study is well explained and experimentally proven using Fourier-transform infrared spectroscopy (FTIR). The mechanism is interesting can be linked to our findings in a way the ion-exchange could also be time–temperature dependent. The present study only provides a baseline for further investigation of the time–temperature dependence of heat capacity enhancement in molten salt nanofluids as this variation may be a result of a combination of multiple phenomena including structure development. In the current study, the lowest rate used is 2 °C min^−1^ as DSC could not be accurately calibrated lower than the value. The study also encourages researchers to explore the effect of even further lower cycling rates and their effect on heart capacity enhancement.

## Conclusion

Molten salt eutectic mixtures show heat capacity enhancement when doped with small concentrations of nanoparticles. The formation of nano-dendritic structures has been proposed as one of the possible mechanisms behind heat capacity enhancements. Therefore, in this study, the time–temperature dependency of these structures was studied experimentally. Several samples of Li_2_CO_3_–K_2_CO_3_ containing 1% Al_2_O_3_ were subjected to different heating and cooling rates (2 to 10 °C min^−1^) in the synthesis. The result shows heat capacity enhancement increases with decreasing heating and cooling rate. Moreover, transmission electron microscopy confirmed nano-dendritic structure is well-developed for samples treated at 2 °C min^−1^ but gradually decreased with increasing heating and cooling rate. Further experimental verification was carried out by the pH variation method reported in the literature.

## Conflicts of interest

There are no conflicts to declare.

## Supplementary Material
